# Piperazine-1,4-diium bis­(hydrogen 2-propyl-1*H*-imidazole-4,5-dicarbox­ylate) monohydrate

**DOI:** 10.1107/S1600536810049822

**Published:** 2010-12-04

**Authors:** Zhu-Qing Gao, Jin-Zhong Gu

**Affiliations:** a School of Chemistry and Biology Engineering, Taiyuan University of Science and Technology, Taiyuan 030021, People’s Republic of China; b College of Chemistry and Chemical Engineering, Lanzhou University, Lanzhou, Gansu 730000, People’s Republic of China

## Abstract

The title compound, C_4_H_12_N_2_^2+^·2C_8_H_9_N_2_O_4_^−^·H_2_O, is a hydrated proton-transfer compound obtained from 2-propyl-1*H*-imidazole-4,5-dicarb­oxy­lic acid and piperazine. The asymmetric unit contains one half-cation, one anion and half a water mol­ecule. There is a centre of inversion at the centre of the cation ring and the water molecule O atom lies on a twofold rotation axis. In the crystal, inter­molecular N—H⋯O and N—H⋯N hydrogen bonds help to construct a three-dimensional framework. Almost symmetrical, intramolecular O—H⋯O inter­actions are also observed.

## Related literature

For the structures and properties of proton-transfer compounds, see: Aghabozorg *et al.* (2006 ▶). For the use of multi-carboxyl­ate heterocyclic acids and piperazine in coord­ination chemistry, see: Murugavel *et al.* (2009 ▶); Sheshmani *et al.* (2006 ▶) and for piperazinium structures, see: Murugavel *et al.* (2009 ▶); Sheshmani *et al.* (2007 ▶). For bond-length data, see: Allen *et al.* (1987 ▶).
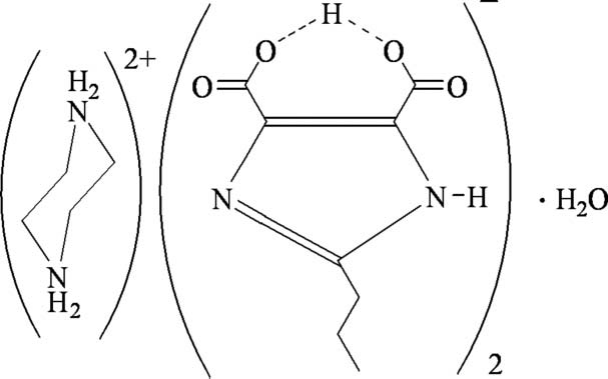


## Experimental

### 

#### Crystal data

C_4_H_12_N_2_^2+^·2C_8_H_9_N_2_O_4_^−^·H_2_O
*M* *_r_* = 500.52
Monoclinic, 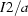

*a* = 11.288 (2) Å
*b* = 15.965 (3) Å
*c* = 14.449 (4) Å
β = 101.296 (12)°
*V* = 2553.6 (10) Å^3^
*Z* = 4
Mo *K*α radiation
μ = 0.10 mm^−1^
*T* = 273 K
0.20 × 0.18 × 0.16 mm

#### Data collection

Bruker SMART CCD area-detector diffractometer
Absorption correction: multi-scan (*SADABS*; Bruker, 1997 ▶) *T*_min_ = 0.980, *T*_max_ = 0.984
6239 measured reflections
2066 independent reflections
1499 reflections with *I* > 2σ(*I*)
*R*_int_ = 0.039

#### Refinement

*R*[*F* ^2^ > 2σ(*F* ^2^)] = 0.050
*wR*(*F* ^2^) = 0.136
*S* = 1.05
2066 reflections
165 parameters
13 restraints
H atoms treated by a mixture of independent and constrained refinement
Δρ_max_ = 0.43 e Å^−3^
Δρ_min_ = −0.21 e Å^−3^

### 

Data collection: *SMART* (Bruker, 1997 ▶); cell refinement: *SAINT* (Bruker, 1997 ▶); data reduction: *SAINT*; program(s) used to solve structure: *SHELXS97* (Sheldrick, 2008 ▶); program(s) used to refine structure: *SHELXL97* (Sheldrick, 2008 ▶); molecular graphics: *SHELXTL* (Sheldrick, 2008 ▶); software used to prepare material for publication: *SHELXTL*.

## Supplementary Material

Crystal structure: contains datablocks I, global. DOI: 10.1107/S1600536810049822/jh2238sup1.cif

Structure factors: contains datablocks I. DOI: 10.1107/S1600536810049822/jh2238Isup2.hkl

Additional supplementary materials:  crystallographic information; 3D view; checkCIF report

## Figures and Tables

**Table 1:** Hydrogen-bond geometry (Å, °)

*D*—H⋯*A*	*D*—H	H⋯*A*	*D*⋯*A*	*D*—H⋯*A*
O3—H2⋯O1	1.19 (3)	1.26 (3)	2.447 (3)	172 (3)
O5—H1*W*⋯O1^i^	0.87	2.24	3.065 (3)	158
N1—H1⋯O2^ii^	0.86	1.94	2.773 (3)	162
N3—H3*A*⋯N2	0.90	1.94	2.820 (3)	165
N3—H3*B*⋯O4^iii^	0.90	1.96	2.826 (3)	161

Symmetry codes: (i) 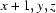; (ii) 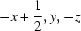; (iii) 
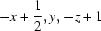
.

**Fig. 1.**
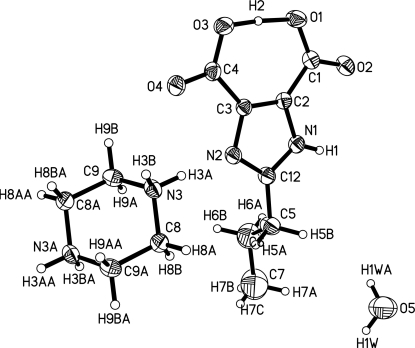
A drawing of the asymmetric unit in the structure of (I), showing the atom-labeling scheme. Displacement ellipsoids are drawn at the 30% probability level.

**Fig. 2.**
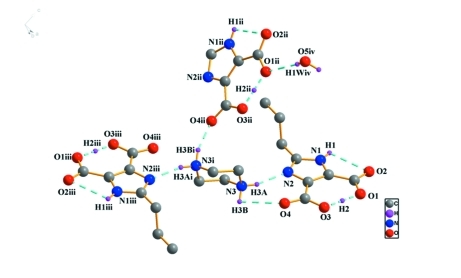
The hydrogen bonds are shown and are depicted by blue dashed lines. Hydrogen atoms that bonded to carbon atoms were omitted for clarity. [Symmetry codes: (i) x, -y + 3/2, z - 1/2; (ii) –x + 1/2, -y + 3/2, -z + 1/2; (iii) x, -y + 3/2, z - 1/2; (iv) –x + 3/2, -y + 3/2, -z + 1/2].

**Fig. 3.**
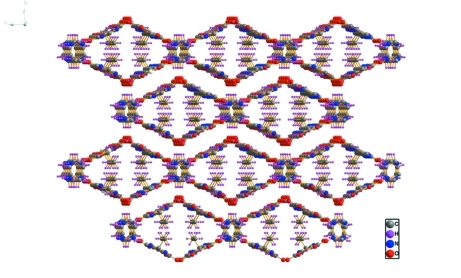
A view along the a axis, showing a three-dimensional framework.

## supplementary crystallographic information

### Comment

t;text-indent:12.0 pt;mso-char-indent-count: 1.0;line-height:200%'>In the past decades, much attention has been focused on the design and synthesis of proton-transfer compounds, owing to their importance in physics, chemistry and biochemistry (Aghabozorg *et al.*, 2006; Allen *et al.*, 1987). Many multi-carboxylate or heterocyclic acids and piperazine are used for this purpose (Murugavel *et al.*, 2009; Sheshmani *et al.*, 2006). In order to extend the investigation, we have prepared the title compound, (I), and report its crystal structure here.

As shown in Fig.1, The asymmetric unit contains one half-cation, one anion and half a water molecule. There is a centre of inversion at the centre of the cation ring and one water molecule lies on a twofold rotation axis. The organic piperazinium dication lies at an inversion centre and adopts a typical chair geometry with normal valence bond lengths (Murugavel *et al.*, 2009) and angles, as observed in the related structures (Sheshmani *et al.*, 2007). The anionic fragment individually has two intramolecular hydrogen bonds, a O–H···O bond between adjacent carboxylate groups and a N–H···O bond between the imidazole ring and the carboxylate group (Fig. 2 and Table 1). In the crystal structure, intermolecular N–H···O and N–H···N hydrogen bonds play an important role in the construction of the three-dimensional framework (Fig. 3).

### Experimental

To a solution of 2-propyl-1*H*-imidazole-4,5-dicarboxylic acid (0.100 g, 0.5 mmol) in water (5 ml) was added an aqueous solution (5 ml) of piperazine (0.089 g, 0.5 mmol). The reactants were sealed in a 25-ml Teflon-lined, stainless-steel Parr bomb. The bomb was heated at 433 K for 3 days. The cool solution yielded single crystals in *ca* 70% yield. Anal. Calcd for C_10_H_16_N_3_O_4.5_: C, 47.99; H, 6.44; N, 16.79. Found: C, 47.61; H, 6.77; N, 16.42.

### Refinement

The free water H atoms attached to oxygen atoms were placed at calculated positions and refined with the riding model, considering the position of oxygen atoms and the quantity of H atoms. The H atoms were placed in geometrically idealized positions, withN–H = 0.86–0.90 Å and C–H = 0.93 Å, and constrained to ride on their respective parent atoms, with *U*iso(H) = 1.2 *U*eq.

### Crystal data

**Table:**

C_4_H_12_N_2_^2+^·2C_8_H_9_N_2_O_4_^−^·H_2_O	*F*(000) = 1064
*M**_r_* = 500.52	*D*_x_ = 1.302 Mg m^−^^3^
Monoclinic, *I*2/*a*	Mo *K*α radiation, λ = 0.71073 Å
*a* = 11.288 (2) Å	Cell parameters from 1047 reflections
*b* = 15.965 (3) Å	θ = 0.0–0.0°
*c* = 14.449 (4) Å	µ = 0.10 mm^−^^1^
β = 101.296 (12)°	*T* = 273 K
*V* = 2553.6 (10) Å^3^	Block, colorless
*Z* = 4	0.20 × 0.18 × 0.16 mm

### Data collection

**Table:**

Bruker SMART CCD area-detector diffractometer	2066 independent reflections
Radiation source: fine-focus sealed tube	1499 reflections with *I* > 2σ(*I*)
graphite	*R*_int_ = 0.039
φ and ω scans	θ_max_ = 24.3°, θ_min_ = 1.9°
Absorption correction: multi-scan (*SADABS*; Bruker, 1997)	*h* = −10→13
*T*_min_ = 0.980, *T*_max_ = 0.984	*k* = −18→17
6239 measured reflections	*l* = −16→16

### Refinement

**Table:**

Refinement on *F*^2^	Primary atom site location: structure-invariant direct methods
Least-squares matrix: full	Secondary atom site location: difference Fourier map
*R*[*F*^2^ > 2σ(*F*^2^)] = 0.050	Hydrogen site location: inferred from neighbouring sites
*wR*(*F*^2^) = 0.136	H atoms treated by a mixture of independent and constrained refinement
*S* = 1.05	*w* = 1/[σ^2^(*F*_o_^2^) + (0.0619*P*)^2^ + 1.5738*P*] where *P* = (*F*_o_^2^ + 2*F*_c_^2^)/3
2066 reflections	(Δ/σ)_max_ = 0.001
165 parameters	Δρ_max_ = 0.43 e Å^−^^3^
13 restraints	Δρ_min_ = −0.21 e Å^−^^3^

### Special details

**Table:**

Geometry. All e.s.d.'s (except the e.s.d. in the dihedral angle between two l.s. planes) are estimated using the full covariance matrix. The cell e.s.d.'s are taken into account individually in the estimation of e.s.d.'s in distances, angles and torsion angles; correlations between e.s.d.'s in cell parameters are only used when they are defined by crystal symmetry. An approximate (isotropic) treatment of cell e.s.d.'s is used for estimating e.s.d.'s involving l.s. planes.
Refinement. Refinement of *F*^2^ against ALL reflections. The weighted *R*-factor *wR* and goodness of fit *S* are based on *F*^2^, conventional *R*-factors *R* are based on *F*, with *F* set to zero for negative *F*^2^. The threshold expression of *F*^2^ > σ(*F*^2^) is used only for calculating *R*-factors(gt) *etc*. and is not relevant to the choice of reflections for refinement. *R*-factors based on *F*^2^ are statistically about twice as large as those based on *F*, and *R*- factors based on ALL data will be even larger.

### Fractional atomic coordinates and isotropic or equivalent isotropic displacement parameters (Å^2^)

**Table:**

	*x*	*y*	*z*	*U*_iso_*/*U*_eq_	
C1	0.0849 (2)	0.71261 (16)	0.09103 (17)	0.0422 (6)	
C2	0.1935 (2)	0.67934 (14)	0.15314 (16)	0.0364 (6)	
C3	0.2144 (2)	0.64165 (14)	0.24013 (16)	0.0363 (6)	
C4	0.1292 (2)	0.62027 (16)	0.30292 (18)	0.0424 (6)	
C5	0.5184 (2)	0.6485 (2)	0.1969 (2)	0.0611 (8)	
H5A	0.5649	0.6478	0.2609	0.073*	
H5B	0.5409	0.6984	0.1661	0.073*	
C6	0.5505 (3)	0.5713 (3)	0.1446 (4)	0.1233 (18)	
H6A	0.5028	0.5710	0.0810	0.148*	
H6B	0.5308	0.5212	0.1765	0.148*	
C7	0.6847 (4)	0.5704 (3)	0.1403 (5)	0.171 (3)	
H7A	0.7039	0.6195	0.1078	0.205*	
H7B	0.7024	0.5213	0.1072	0.205*	
H7C	0.7320	0.5699	0.2032	0.205*	
C8	0.5499 (2)	0.57768 (16)	0.47554 (19)	0.0483 (7)	
H8A	0.5643	0.6370	0.4872	0.058*	
H8B	0.5864	0.5617	0.4227	0.058*	
C9	0.3930 (2)	0.47110 (16)	0.43879 (17)	0.0456 (7)	
H9A	0.4231	0.4509	0.3845	0.055*	
H9B	0.3062	0.4627	0.4267	0.055*	
C12	0.3877 (2)	0.65313 (16)	0.20022 (17)	0.0429 (6)	
H2	−0.004 (2)	0.6647 (18)	0.195 (2)	0.103 (11)*	
H1W	0.8140	0.7443	0.0209	0.31 (5)*	
N1	0.30493 (17)	0.68560 (13)	0.12993 (13)	0.0410 (5)	
H1	0.3196	0.7069	0.0787	0.049*	
N2	0.33568 (18)	0.62552 (13)	0.26903 (14)	0.0426 (5)	
N3	0.42026 (17)	0.56200 (13)	0.45216 (14)	0.0426 (5)	
H3A	0.3883	0.5897	0.3989	0.051*	
H3B	0.3856	0.5818	0.4988	0.051*	
O1	−0.01641 (15)	0.70340 (13)	0.11795 (13)	0.0582 (6)	
O2	0.09473 (15)	0.74761 (12)	0.01667 (12)	0.0510 (5)	
O3	0.01637 (15)	0.63600 (13)	0.27299 (13)	0.0566 (5)	
O4	0.16861 (16)	0.58907 (12)	0.38055 (12)	0.0537 (5)	
O5	0.7500	0.7747 (3)	0.0000	0.170 (3)	

### Atomic displacement parameters (Å^2^)

**Table:**

	*U*^11^	*U*^22^	*U*^33^	*U*^12^	*U*^13^	*U*^23^
C1	0.0499 (16)	0.0430 (15)	0.0338 (14)	0.0040 (12)	0.0083 (12)	−0.0025 (12)
C2	0.0403 (13)	0.0379 (14)	0.0322 (13)	0.0010 (10)	0.0101 (10)	−0.0008 (10)
C3	0.0388 (13)	0.0379 (14)	0.0331 (13)	0.0015 (10)	0.0092 (10)	0.0022 (10)
C4	0.0480 (15)	0.0419 (15)	0.0390 (15)	0.0021 (11)	0.0124 (12)	0.0047 (12)
C5	0.0471 (17)	0.088 (2)	0.0514 (17)	0.0117 (15)	0.0188 (14)	0.0195 (16)
C6	0.090 (3)	0.089 (3)	0.215 (5)	0.042 (2)	0.089 (3)	0.042 (3)
C7	0.130 (4)	0.149 (5)	0.268 (7)	0.062 (4)	0.126 (5)	0.072 (5)
C8	0.0438 (15)	0.0455 (16)	0.0572 (17)	0.0039 (12)	0.0138 (13)	0.0078 (13)
C9	0.0408 (14)	0.0511 (17)	0.0438 (16)	0.0050 (12)	0.0055 (11)	−0.0065 (12)
C12	0.0437 (14)	0.0530 (16)	0.0335 (14)	0.0049 (12)	0.0110 (11)	0.0054 (12)
N1	0.0469 (12)	0.0483 (13)	0.0307 (11)	0.0027 (10)	0.0150 (9)	0.0058 (9)
N2	0.0437 (12)	0.0494 (13)	0.0366 (12)	0.0059 (9)	0.0127 (9)	0.0089 (9)
N3	0.0448 (12)	0.0479 (13)	0.0354 (11)	0.0108 (9)	0.0090 (9)	0.0058 (9)
O1	0.0429 (11)	0.0824 (15)	0.0487 (12)	0.0088 (9)	0.0075 (9)	0.0143 (10)
O2	0.0610 (12)	0.0601 (12)	0.0334 (10)	0.0150 (9)	0.0132 (9)	0.0053 (8)
O3	0.0427 (11)	0.0766 (14)	0.0527 (12)	0.0031 (9)	0.0146 (9)	0.0180 (10)
O4	0.0540 (11)	0.0682 (13)	0.0426 (11)	0.0087 (9)	0.0189 (9)	0.0200 (9)
O5	0.084 (3)	0.089 (3)	0.315 (8)	0.000	−0.015 (4)	0.000

### Geometric parameters (Å, °)

**Table:**

C1—O2	1.235 (3)	C7—H7C	0.9600
C1—O1	1.286 (3)	C8—N3	1.458 (3)
C1—C2	1.469 (3)	C8—C9^i^	1.497 (3)
C2—N1	1.368 (3)	C8—H8A	0.9700
C2—C3	1.372 (3)	C8—H8B	0.9700
C3—N2	1.375 (3)	C9—N3	1.488 (3)
C3—C4	1.486 (3)	C9—C8^i^	1.497 (3)
C4—O4	1.228 (3)	C9—H9A	0.9700
C4—O3	1.287 (3)	C9—H9B	0.9700
C5—C12	1.487 (3)	C12—N2	1.325 (3)
C5—C6	1.526 (5)	C12—N1	1.342 (3)
C5—H5A	0.9700	N1—H1	0.8600
C5—H5B	0.9700	N3—H3A	0.9000
C6—C7	1.528 (5)	N3—H3B	0.9000
C6—H6A	0.9700	O1—H2	1.26 (3)
C6—H6B	0.9700	O3—H2	1.19 (3)
C7—H7A	0.9600	O5—H1W	0.8739
C7—H7B	0.9600		
			
O2—C1—O1	123.5 (2)	H7B—C7—H7C	109.5
O2—C1—C2	119.2 (2)	N3—C8—C9^i^	110.6 (2)
O1—C1—C2	117.3 (2)	N3—C8—H8A	109.5
N1—C2—C3	104.8 (2)	C9^i^—C8—H8A	109.5
N1—C2—C1	121.4 (2)	N3—C8—H8B	109.5
C3—C2—C1	133.8 (2)	C9^i^—C8—H8B	109.5
C2—C3—N2	110.1 (2)	H8A—C8—H8B	108.1
C2—C3—C4	130.1 (2)	N3—C9—C8^i^	110.8 (2)
N2—C3—C4	119.8 (2)	N3—C9—H9A	109.5
O4—C4—O3	122.9 (2)	C8^i^—C9—H9A	109.5
O4—C4—C3	119.3 (2)	N3—C9—H9B	109.5
O3—C4—C3	117.8 (2)	C8^i^—C9—H9B	109.5
C12—C5—C6	112.9 (3)	H9A—C9—H9B	108.1
C12—C5—H5A	109.0	N2—C12—N1	110.5 (2)
C6—C5—H5A	109.0	N2—C12—C5	126.6 (2)
C12—C5—H5B	109.0	N1—C12—C5	122.9 (2)
C6—C5—H5B	109.0	C12—N1—C2	108.89 (19)
H5A—C5—H5B	107.8	C12—N1—H1	125.6
C5—C6—C7	111.2 (4)	C2—N1—H1	125.6
C5—C6—H6A	109.4	C12—N2—C3	105.7 (2)
C7—C6—H6A	109.4	C8—N3—C9	111.74 (18)
C5—C6—H6B	109.4	C8—N3—H3A	109.3
C7—C6—H6B	109.4	C9—N3—H3A	109.3
H6A—C6—H6B	108.0	C8—N3—H3B	109.3
C6—C7—H7A	109.5	C9—N3—H3B	109.3
C6—C7—H7B	109.5	H3A—N3—H3B	107.9
H7A—C7—H7B	109.5	C1—O1—H2	112.1 (11)
C6—C7—H7C	109.5	C4—O3—H2	112.9 (12)
H7A—C7—H7C	109.5		

Symmetry codes: (i) −*x*+1, −*y*+1, −*z*+1.

### Hydrogen-bond geometry (Å, °)

**Table:**

*D*—H···*A*	*D*—H	H···*A*	*D*···*A*	*D*—H···*A*
O3—H2···O1	1.19 (3)	1.26 (3)	2.447 (3)	172 (3)
O5—H1W···O1^ii^	0.87	2.24	3.065 (3)	158.
N1—H1···O2^iii^	0.86	1.94	2.773 (3)	162.
N3—H3A···N2	0.90	1.94	2.820 (3)	165.
N3—H3B···O4^iv^	0.90	1.96	2.826 (3)	161.

Symmetry codes: (ii) *x*+1, *y*, *z*; (iii) −*x*+1/2, *y*, −*z*; (iv) −*x*+1/2, *y*, −*z*+1.
